# Artificial intelligence integration in surgery through hand and instrument tracking: a systematic literature review

**DOI:** 10.3389/fsurg.2025.1528362

**Published:** 2025-02-26

**Authors:** Kivanc Yangi, Thomas J. On, Yuan Xu, Arianna S. Gholami, Jinpyo Hong, Alexander G. Reed, Pravarakhya Puppalla, Jiuxu Chen, Jonathan A. Tangsrivimol, Baoxin Li, Marco Santello, Michael T. Lawton, Mark C. Preul

**Affiliations:** ^1^ The Loyal and Edith Davis Neurosurgical Research Laboratory, Department of Neurosurgery, Barrow Neurological Institute, St. Joseph’s Hospital and Medical Center, Phoenix, AZ, United States; ^2^ School of Computing and Augmented Intelligence, Arizona State University, Tempe, AZ, United States; ^3^ School of Biological and Health Systems Engineering, Arizona State University, Tempe, AZ, United States

**Keywords:** artificial intelligence, deep learning, education, hand tracking, instrument tracking, machine learning, surgery, surgical motion

## Abstract

**Objective:**

This systematic literature review of the integration of artificial intelligence (AI) applications in surgical practice through hand and instrument tracking provides an overview of recent advancements and analyzes current literature on the intersection of surgery with AI. Distinct AI algorithms and specific applications in surgical practice are also examined.

**Methods:**

An advanced search using medical subject heading terms was conducted in Medline (via PubMed), SCOPUS, and Embase databases for articles published in English. A strict selection process was performed, adhering to PRISMA guidelines.

**Results:**

A total of 225 articles were retrieved. After screening, 77 met inclusion criteria and were included in the review. Use of AI algorithms in surgical practice was uncommon during 2013–2017 but has gained significant popularity since 2018. Deep learning algorithms (*n* = 62) are increasingly preferred over traditional machine learning algorithms (*n* = 15). These technologies are used in surgical fields such as general surgery (*n* = 19), neurosurgery (*n* = 10), and ophthalmology (*n* = 9). The most common functional sensors and systems used were prerecorded videos (*n* = 29), cameras (*n* = 21), and image datasets (*n* = 7). The most common applications included laparoscopic (*n* = 13), robotic-assisted (*n* = 13), basic (*n* = 12), and endoscopic (*n* = 8) surgical skills training, as well as surgical simulation training (*n* = 8).

**Conclusion:**

AI technologies can be tailored to address distinct needs in surgical education and patient care. The use of AI in hand and instrument tracking improves surgical outcomes by optimizing surgical skills training. It is essential to acknowledge the current technical and social limitations of AI and work toward filling those gaps in future studies.

## 1 Introduction

The current paradigm shift in medicine involves technological advancements aimed at optimizing patient care tasks through research and collaboration. Artificial intelligence (AI) and machine learning (ML) algorithms are applicable in precision medicine, allowing integrative and personalized approaches to solve complex medical problems (1).

AI applications in surgical practice, mainly associated with hand tracking, have evolved significantly over time. Integrating AI technologies into surgical practice began attracting attention in the early 2010s; however, the use of AI algorithms for hand and instrument tracking is a more recent advancement (2). These technologies have been explored across various surgical specialties, including ophthalmology; plastic, reconstructive and aesthetic surgery; endocrine surgery; cardiac surgery; and general surgery (3–9). The main goals of using AI algorithms in surgical practice include enhancing surgical safety, decreasing the duration of time-consuming procedures, improving minimally invasive techniques, and facilitating the training of junior surgeons and students through surgical simulation education (10–12).

In recent years, ML technologies have also been used for disease detection and prevention, radiological interpretations, and improving surgical precision (13–16). The focus of this study was on the role of AI and ML in analyzing surgical precision. ML is often directed toward generating hand-tracking and motion-tracking solutions for the best possible postsurgical outcomes. AI and ML technologies can assess and improve human performance beyond conventional standards, particularly in surgical tasks.

A systematic review of the current literature on AI applications in surgical practice, with a particular focus on hand and instrument tracking, was conducted. AI technologies and their applications in various surgical procedures were explored. It also examined the numerous types of sensors employed in these processes. Finally, the analysis addressed the various AI algorithms, their application in distinct surgical areas via different types of sensors, and the use of AI for hand and instrument tracking in surgical practice, including the associated challenges and future directions.

## 2 Materials and methods

### 2.1 Search strategy

Systematic searches of the Medline (via PubMed), SCOPUS, and Embase databases were conducted in July 2024 without time restrictions, using the following keywords: [(Surgical motion) OR (Hand tracking) OR (instrument tracking) OR (surgical gesture) OR (instrument detection) OR (tool movement) OR (instrument movement) OR (MoCap Motion Capture) OR (Motion Capture, MoCap) OR (Biomechanical Movement Capture) OR (Movement Capture, Biomechanical) OR (Optical Motion Capture) OR (Motion Capture, Optical) OR (Magnetic Motion Capture) OR (Motion Capture, Magnetic)] AND [(Machine Learning) OR (Learning, Machine) OR (Transfer Learning) OR (Learning, Transfer) OR (Deep Learning) OR (Learning, Deep) OR (Hierarchical Learning) OR (Learning, Hierarchical) OR (Intelligence, Artificial) OR (Computer Reasoning) OR (Reasoning, Computer) OR [AI (Artificial Intelligence)] OR (Machine Intelligence) OR (Intelligence, Machine) OR (Computational Intelligence) OR (Intelligence, Computational) OR (Computer Vision Systems) OR (Computer Vision System) OR (System, Computer Vision) OR (Systems, Computer Vision) OR (Vision System, Computer) OR (Vision Systems, Computer) OR [Knowledge Acquisition (Computer)] OR [Acquisition, Knowledge (Computer)] OR [Knowledge Representation (Computer)] OR [Knowledge Representations (Computer)] OR [Representation, Knowledge (Computer)] AND [(Surgery) OR (Surgical Procedures) OR (Procedures, Surgical) OR (Procedure, Surgical) OR (Surgical Procedure) OR (Operative Procedures) OR (Operative Procedure) OR (Procedure, Operative) OR (Procedures, Operative) OR (Operative Surgical Procedure) OR (Procedure, Operative Surgical) OR (Procedures, Operative Surgical) OR (Surgical Procedure, Operative) OR (Operative Procedures) OR (Operations) OR (Invasive Procedures) OR (Operative Therapy) OR (Preoperative Procedures) OR (Intraoperative Procedures) OR (Peroperative Procedures) OR (Perioperative Procedures)]. These medical subject heading terms were linked with Boolean operators “AND” and “OR” to maximize comprehensiveness.

### 2.2 Eligibility criteria

Our inclusion criteria focused on original research articles that used AI methods in surgical practice, specifically through the application of various sensors for hand or instrument tracking during surgical procedures. Articles were excluded if they lacked any of the 3 key components: AI systems, application in surgical procedures, or tracking of hands or instruments (Table 1). Additionally, review articles, editorials, errata, retracted articles, and studies without accessible full texts or those not available in English were excluded.

**Table 1:** Inclusion and exclusion criteria.

Inclusion criteria	Exclusion criteria
Original research articles	Review articles, editorials, errata, retracted papers
Articles applying AI systems to surgical practice through hand or instrument tracking.	Full text unavailable
	Non-English language
	Not hand or instrument tracking
	Not applying to surgical practice

Articles that did not involve any of the 3 components (AI systems, application in surgical procedures, or tracking of hands or instruments) were excluded.

### 2.3 Study selection

Our search terms and articles were filtered by title or abstract. Only articles written in the English language were screened. Duplications were excluded. Three independent reviewers (A.S.G., J.H., K.Y.) screened the articles using the Rayaan platform (17). A strict selection process was performed, adhering to the Preferred Reporting Items for Systematic Reviews and Meta-Analysis (PRISMA) guidelines (18).

A manual search was also conducted to explore any additional relevant articles, and the reference lists of all included articles were examined by 2 independent reviewers (J.H. and A.S.G.), as recommended by systematic review manuals (19). After the screening, disagreements were discussed, and all reviewers (T.J.O., A.S.G., J.H., K.Y., P.P., A.G.R.) reached a consensus to include 77 articles in the study.

## 3 Results

**Figure 1:**
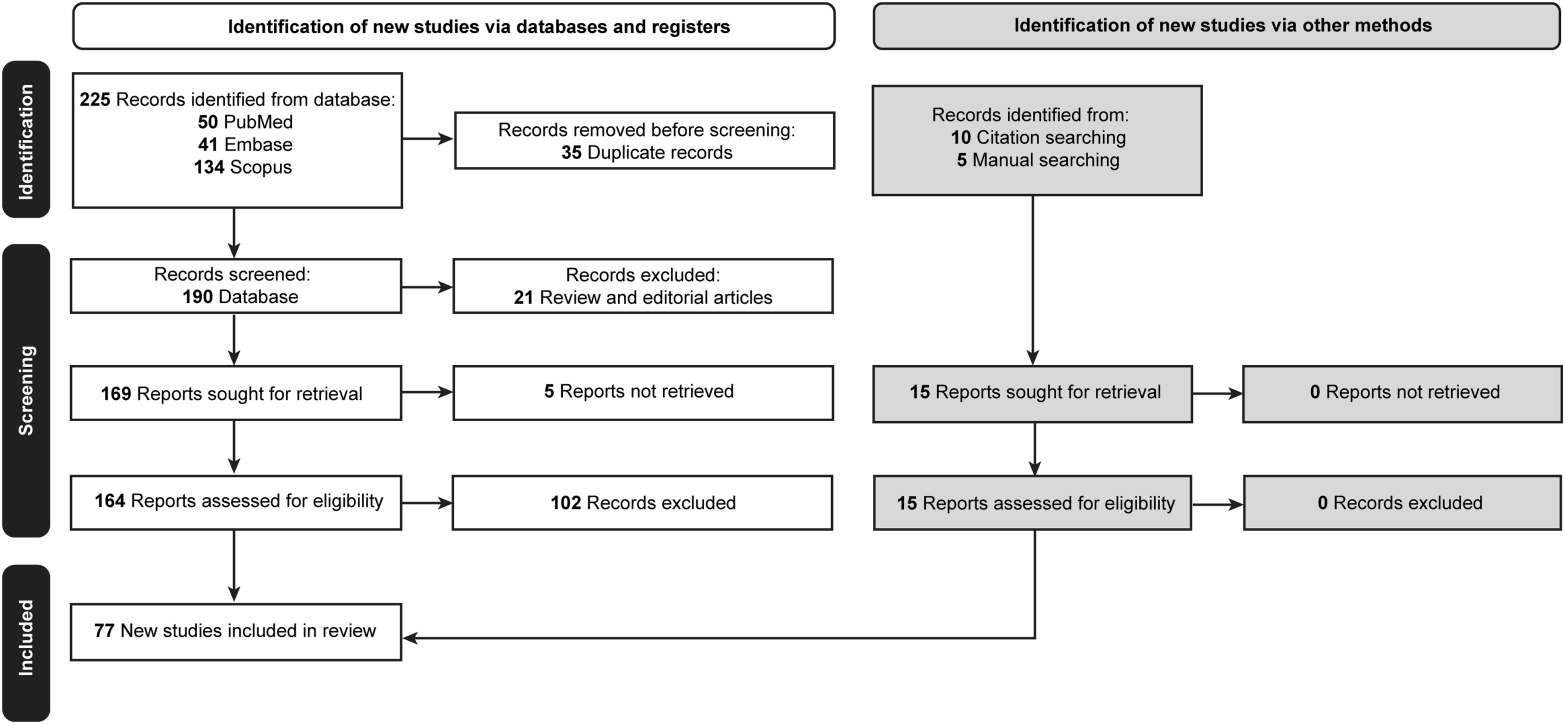
Flow diagram documenting the study selection process. Used with permission from Barrow Neurological Institute, Phoenix, Arizona.

Initially, 225 articles were retrieved. Thirty-five duplicated papers, 21 review articles and editorials, and 5 articles for which the full text was unavailable were excluded. Of the 164 remaining articles, 102 did not meet the inclusion criteria and were excluded. A reference-checking method was performed, and 10 additional articles were found that were within the scope of the study and included. A manual search method was also conducted, and 5 articles were found to be relevant and included in the study. Thus, after screening, a total of 77 articles were found to be eligible for the study and were included (Table 2) (2–12, 20–84). The selection process is documented in Figure 1 (85).

**Table 2:** Summary of studies focused on applying artificial intelligence algorithms to surgical practice through hand and instrument tracking.

Study (year)	Machine learning method	Sensor or system used	Surgical field	Procedure
AbuSneineh and Seales (2013) (20)	Traditional machine learning	ECG monitor, eye trackers, cameras	Nonspecific surgery	Minimally invasive surgery; pegboard ring transfer task
Ryu et al. (2013) (2)	Traditional machine learning	Endoscopic vision-based tracking	General surgery	Laparoscopic surgery
Cavallo et al. (2014) (21)	Traditional machine learning	Optical movement detector, sensor instrument data logger module	General surgery	Laparoscopic procedures
Partridge et al. (2014) (22)	Traditional machine learning	Simulator camera and colored bands placed around the distal instrument shafts	General surgery	Surgical simulation training (threading string through hoops)
Dayak and Cevik (2015) (12)	Traditional machine learning	Logitech HD C525 webcam	Nonspecific surgery	Minimally invasive surgery; surgical simulation training
Sahu et al. (2016) (23)	Traditional machine learning	Laparoscopic images	General surgery	Robotized laparoscopic minimally invasive surgery
Chen et al. (2017) (24)	Deep learning	Line segment detector	Nonspecific surgery	Robotic minimally invasive surgery
Mishra et al. (2017) (25)	Deep learning	Retinal microscopy instrument tracking dataset	Ophthalmology	Retinal microsurgery
Zisimopoulos et al. (2017) (26)	Deep learning	Video data and simulation	Ophthalmology	Cataract surgery
Du et al. (2018) (27)	Deep learning	Endoscopic and microscopic datasets	Nonspecific surgery	Articulated endoscopic surgical instrument tracking
Kil et al. (2018) (28)	Deep learning	Cameras	Nonspecific surgery	Assessment of suturing skill
Richey et al. (2018) (29)	Traditional machine learning	Bumblebee XB3 and Grasshopper stereo camera system; Polaris Vicra	General surgery	Lumpectomy
Wang and Fey (2018) (30)	Deep learning	da Vinci surgical system	Nonspecific surgery	Minimally invasive surgery; robotic surgery, suturing, needle passing, and knot tying
Wesierski and Jezierska (2018) (31)	Traditional machine learning	Cameras; videos	Nonspecific surgery	Minimally invasive surgery; detection and pose estimation during robotic surgery
Zhang et al. (2018) (32)	Deep learning	Video recordings	Urology	Prostatectomy
Azari et al. (2019) (33)	Traditional machine learning	GZ-E300 JVC camcorders	Nonspecific surgery	Simple interrupted and runner subcuticular suturing
Du et al. (2019) (34)	Traditional machine learning	Videos	General surgery	Minimally invasive surgery
El-Haddad et al. (2019) (35)	Deep learning	Volumetric OCT data	Ophthalmology	Ophthalmic surgery
Kowalewski et al. (2019) (36)	Traditional machine learning	Myo armband; NDI Polaris camera	General surgery	Laparoscopic suturing and knot-tying tasks
Funke et al. (2019) (37)	Deep learning	Virtual camera programmed to mimic Intel RealSense D435	Nonspecific surgery	Minimally invasive surgery; suturing on da Vinci machine
Qiu et al. (2019) (38)	Deep learning	m2cai16-tool based dataset	Nonspecific surgery	Minimally invasive robot-assisted surgery
Tang et al. (2019) (39)	Deep learning	Custom 1,060 nm swept-source OCT engine to perform SPECT imaging	Ophthalmology	Intraoperative optical coherence tomography
Winkler-Schwartz et al. (2019) (40)	Traditional machine learning	High-fidelity neurosurgical simulator	Neurosurgery	Tumor resection in virtual reality
Zhao et al. (2019) (41)	Deep learning	Videos	General surgery	Robot-assisted minimally invasive surgery
Zia et al. (2019) (42)	Deep learning	Videos	Urology	Robotic-assisted radical prostatectomies
Khalid et al. (2020) (43)	Deep learning	Videos	Nonspecific surgery	Knot tying; suturing; needle passing
Lee et al. (2020) (7)	Deep learning	Video recordings	General surgery	Thyroidectomy; robotic surgery
Leong-Hoi et al. (2020) (44)	Deep learning	Depth camera	General surgery	Laparoscopic mini-invasive surgery
Morita et al. (2020) (45)	Deep learning	High-resolution video recordings	Ophthalmology	Cataract surgery
Sivarasa and Jerew (2020) (46)	Deep learning	Videos	Nonspecific surgery	Endoscopic procedures
Tang et al. (2020) (47)	Deep learning	OCT and SER images	Ophthalmology	Intraoperative OCT
Yu et al. (2020) (48)	Deep learning	Video dataset	Nonspecific surgery	da Vinci surgical system doing 6 different surgical tasks
Zhang et al. (2020) (4)	Deep learning	Preselected video footage from YouTube	General surgery	Laparoscopic surgery; breast, GI, and head and neck surgery
Zhang and Gao (2020) (49)	Deep learning	Laparoscopic camera	General surgery	Laparoscopic surgery
Cheng et al. (2021) (50)	Deep learning	Magnetic endoscope	Thoracic surgery	Video-assisted thoracoscopy surgery
Deng et al. (2021) (51)	Deep learning	Image dataset	Otolaryngology	Identification of electrocautery surgical instruments from 20 open-neck procedures
Garcia Nespolo et al. (2021) (52)	Deep learning	Video recordings	Ophthalmology	Cataract surgery
Hein et al. (2021) (53)	Deep learning	RGB frames	Neurosurgery	Spine surgery
Sani et al. (2021) (5)	Deep learning	Inertial measurement units, strain gauges, Polaris Spectra optical motion capture	Cardiac surgery	Mock heart microsurgery procedure
Agarwal et al. (2022) (54)	Deep learning	Videos	Nonspecific surgery	Suturing task
De Backer et al. (2022) (55)	Deep learning	Annotated video frames from robotic surgeries	Urology	Robot-assisted partial nephrectomy and robot-assisted minimally invasive esophagectomy
Despinoy et al. (2016) (56)	Deep learning	Videos	Nonspecific surgery	Surgical hand gesture recognition
Doughty and Ghugre (2022) (57)	Deep learning	Low-latency video	Nonspecific surgery	Surgery involving rigid surgical drill
Ebina et al. (2022) (58)	Traditional machine learning	Motion capture system	General surgery	Automatic assessment of laparoscopic surgery skills
Ebina et al. (2022) (59)	Traditional machine learning	Motion capture system	Urology	Tissue dissection task and parenchymal suturing task
Gazis et al. (2022) (60)	Deep learning	Video dataset	General surgery	Laparoscopy; 2 basic laparoscopic tasks (peg transfer and knot tying)
Huang et al. (2022) (61)	Deep learning	Images; intelligent flexible endoscope system	Nonspecific surgery	Minimally invasive surgery
Kil et al. (2022) (62)	Deep learning	Cameras (Firefly MV USB 2.0, Point Grey Research Inc., British Columbia, Canada) and InertiaCube4 sensor	Nonspecific surgery	Assessment of open surgery suturing skill
Müller et al. (2022) (63)	Deep learning	Intel RealSense D435i RGB camera	General surgery	Telestration for laparoscopic surgery
Pangal et al. (2022) (64)	Traditional machine learning	Intraoperative video recordings	Neurosurgery	Vascular injury control during endonasal surgery
Soleymani et al. (2022) (65)	Deep learning	JIGSAWS dataset; a dual sparse coding algorithm	Nonspecific surgery	Interpreting surgical trajectories
Tang et al. (2022) (3)	Deep learning	SECTR system; OCT	Ophthalmology	Ophthalmic microsurgery
Yamazaki et al. (2022) (66)	Deep learning	Video dataset	General surgery	Laparoscopic infrapyloric lymphadenectomy and suprapancreatic lymphadenectomy during laparoscopic gastrectomy for gastric cancer
Yibulayimu et al. (2022) (67)	Traditional machine learning	Six-dimensional force sensor embedded in liposuction handle; OptiTrack camera	Plastic surgery	Liposuction
Baldi et al. (2023) (6)	Deep learning	Zeiss Lumera microscope	Ophthalmology	Vitreoretinal surgery
Balu et al. (2023) (68)	Deep learning	Videos	Neurosurgery	Detection of surgical instruments in endoscopic endonasal carotid artery laceration repair in a perfused cadaveric simulator
Burton et al. (2023) (69)	Deep learning	RGB camera	Nonspecific surgery	Six-degree-of-freedom pose estimation of a representative surgical instrument in RGB scenes
De Backer et al. (2023) (70)	Deep learning	Endoscopic video data	Urology	Robot-assisted partial nephrectomy and kidney transplant
Gonzalez-Romo et al. (2023) (11)	Deep learning	Zeiss Pentero microscope; GoPro camera	Neurosurgery	Microvascular anastomosis simulation
Kiyasseh et al. (2023) (71)	Deep learning	Videos	Nonspecific surgery	Gesture recognition and skill assessment in robotic-assisted surgery
Kögl et al. (2023) (8)	Deep learning	Single monocular RGB camera	Neurosurgery	Burr hole placement
Lin et al. (2023) (72)	Deep learning	Cameras	General surgery	Robot-assisted laparoscopic surgery
Louis et al. (2023) (73)	Deep learning	Video dataset	Nonspecific surgery	Hand-pose estimation
Luijten et al. (2023) (74)	Deep learning	Structured-light 3D scanners	Oral surgery	Oral and maxillofacial surgery
Ran et al. (2023) (75)	Deep learning	Image dataset	Orthopedic surgery	Surgical instrument detection
Yang et al. (2023) (76)	Deep learning	Videos	General surgery	Peritoneal closure
Yuan et al. (2023) (9)	Deep learning	High-resolution microscope camera	Otolaryngology	Mastoidectomy and cochlear implant
Zheng and Liu (2023) (77)	Deep learning	YOLOv7	Nonspecific surgery	Minimally invasive surgery
Badilla-Solórzano et al. (2024) (78)	Deep learning	Annotated image dataset	Oral surgery	Wisdom teeth extraction surgery
Balu et al. (2024) (79)	Deep learning	Videos	Neurosurgery	Minimally invasive spine surgery; CSF leak; durotomy repair
Jurosch et al. (2024) (80)	Deep learning	Laparoscopic surgical videos recorded via 2D cameras	General surgery	Surgical instrument location detection
Kil et al. (2024) (81)	Deep learning	Cameras	Nonspecific surgery	Assessment of open surgery suturing skill
Kim et al. (2024) (82)	Deep learning	Image dataset	Neurosurgery	Estimation of accurate localization of spinal fixation instruments
Raymond et al. (2024) (83)	Deep learning	Surgical microscope	Otolaryngology	Mastoidectomy
Simoens et al. (2024) (10)	Deep learning	Endoscopic camera	Urology	Robotic surgery, exercise training
Sugiyama et al. (2024) (84)	Deep learning	Videos	Neurosurgery	Microvascular anastomosis training
Viviers et al. (2024) (85)	Deep learning	External optical camera	Neurosurgery	Spine surgery

ECG, electrocardiogram; GI, gastrointestinal; HD, high definition; JIGSAWS, Johns Hopkins University–Intuitive Surgical Inc. Gesture and Skill Assessment Working Set; OCT, optic coherence tomography; RGB, red-green-blue; SECTR, spectrally encoded coherence tomography and reflectometry; SER, spectrally encoded reflectometry; SPECT, single-photon emission computed tomography.

**Figure 2:**
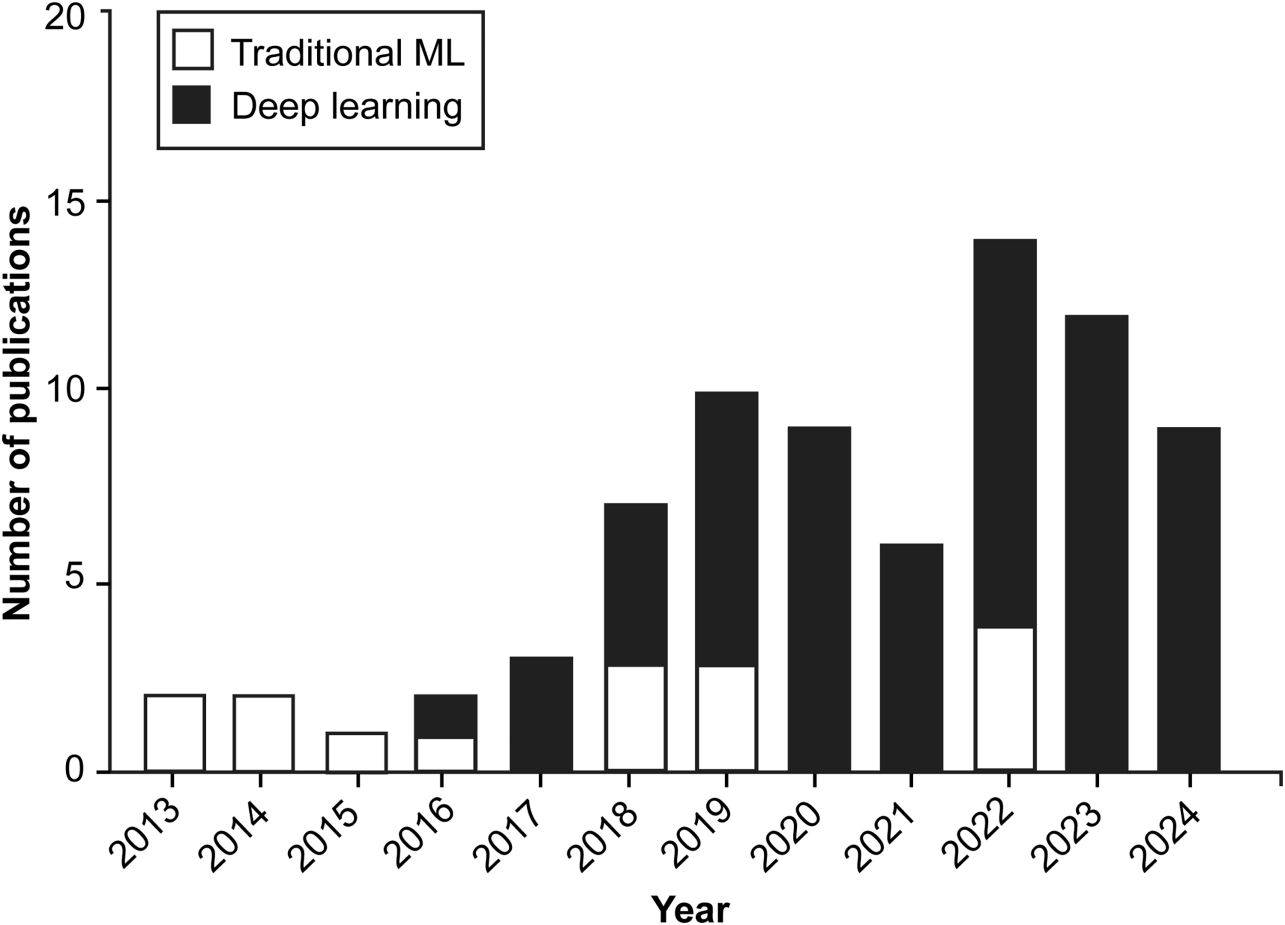
Bar chart illustrating the yearly distribution of publications exploring the application of machine learning (ML) and artificial intelligence algorithms in surgical practice through hand and instrument tracking. Since 2018, there has been a shift in the preferred types of ML algorithms used in these studies, with deep learning techniques gaining prominence over traditional machine learning methods. Although applications of these technologies to surgical practice through hand and instrument tracking were relatively sparse between 2013 and 2017, research interest in these technologies has increased substantially since 2018. *Used with permission from Barrow Neurological Institute, Phoenix, Arizona*.

According to our study's findings, applying AI algorithms in hand and instrument tracking was an exciting but relatively little-studied topic between 2013 and 2017, whereas interest in this area has increased significantly since 2018, with a marked increase in research activity in the 2020s (Figure 2).

**Figure 3:**
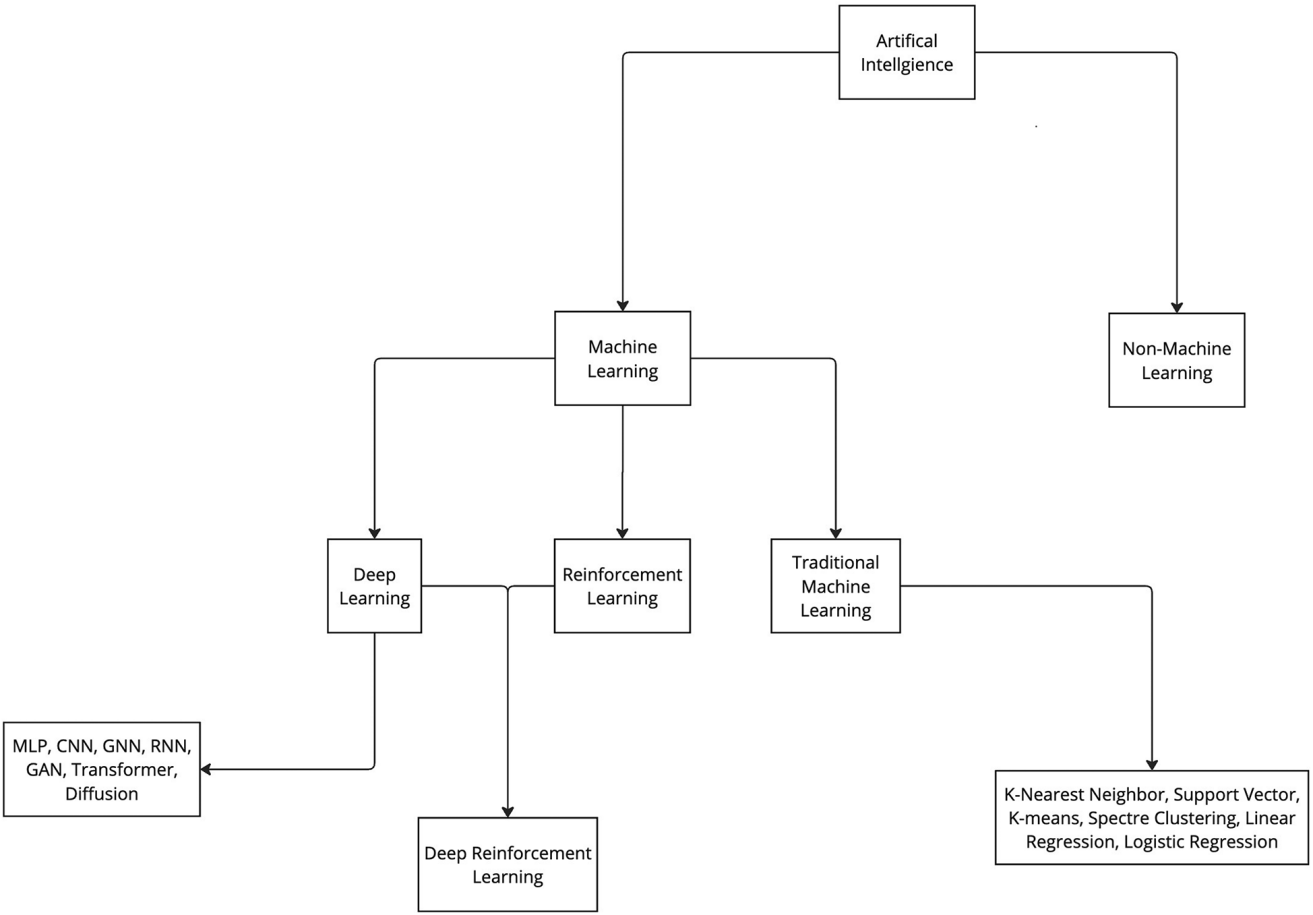
Categorization of artificial intelligence and machine learning. Artificial intelligence algorithms can be categorized into 2 groups: machine learning and non-machine learning. ML algorithms can be further categorized in 3 main groups: deep learning, reinforcement learning, and traditional machine learning. Furthermore, all these groups can be further divided into various subgroups. CNN, convolutional neural network; GAN, generative adversarial network; GNN, graph neural network; MLP, multilayer perceptron; RNN, recurrent neural network. *Used with permission from Barrow Neurological Institute, Phoenix, Arizona*.

Various ML algorithms were used in these studies. For clarity, these algorithms can be categorized into deep learning and traditional ML (non–deep learning) algorithms (Figure 3). This review indicates that deep learning algorithms are significantly more popular than traditional ML algorithms. Specifically, 62 articles employed deep learning, whereas only 15 studies used traditional ML algorithms (Figure 2).

**Figure 4:**
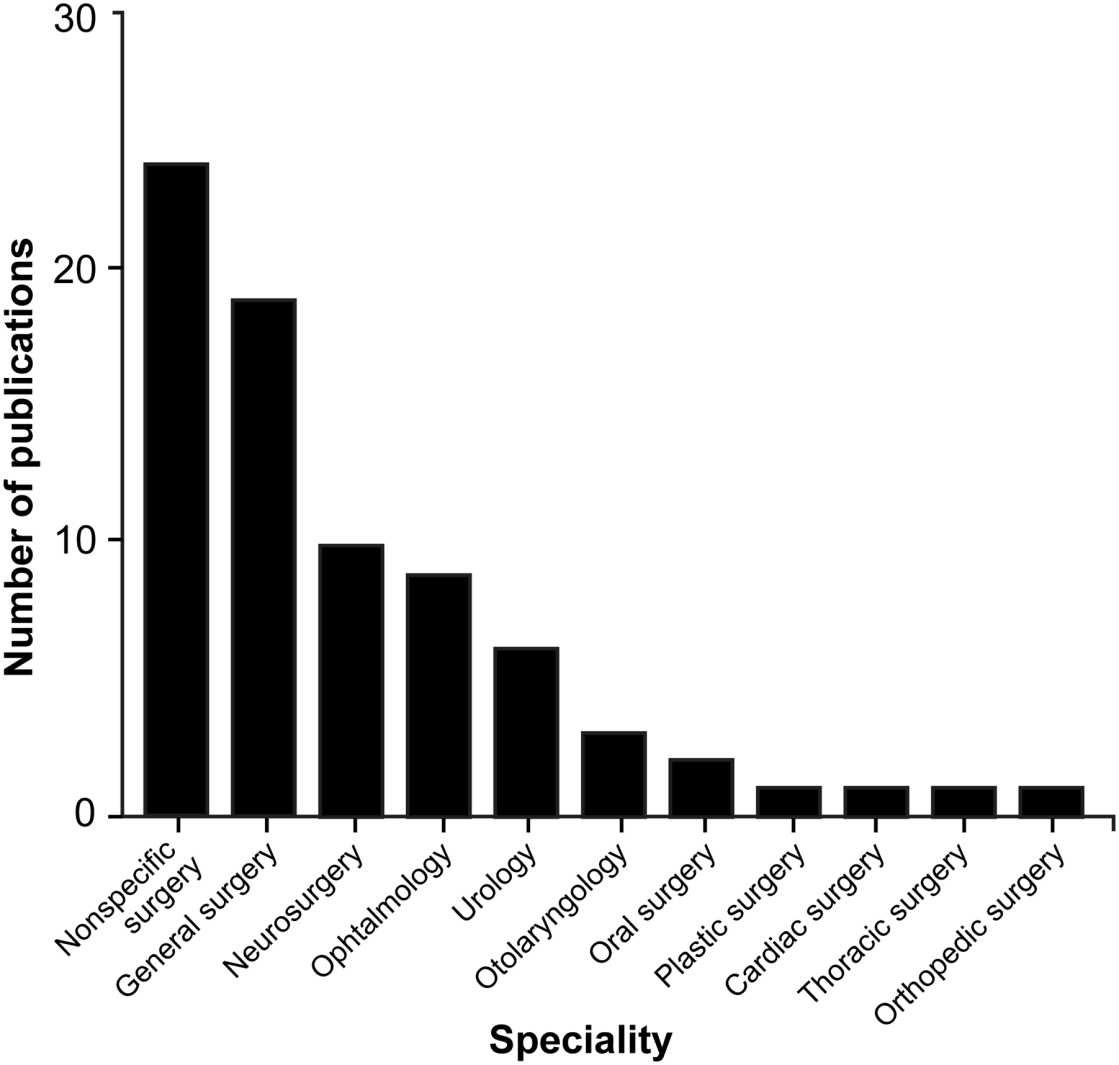
Bar chart showing the surgical fields in which machine learning (ML) algorithms are most commonly applied through hand and instrument tracking methods. As illustrated in the chart, the use of ML algorithms in hand and instrument tracking methods is most widespread in general surgery, neurosurgery, and ophthalmology. However, research has also begun in various surgical fields, including oral surgery, orthopedics, plastic surgery, otolaryngology, urology, cardiac surgery, and thoracic surgery. The nonspecific surgery group includes studies focused on surgical simulation training, hand pose estimation, suturing, knot tying, and basic laparoscopic and endoscopic training, which do not belong to any particular surgical field. *Used with permission from Barrow Neurological Institute, Phoenix, Arizona*.

Examining the different functional sensors used in ML applications within surgical practice reveals a lack of uniformity, with a wide range of sensors being used. However, image datasets (*n* = 7), videos (*n* = 29), and camera recordings (*n* = 21) are among the main systems used to collect information or train the algorithms (Table 2). ML algorithms are most extensively studied in general surgery (*n* = 19), neurosurgery (*n* = 10), and ophthalmology (*n* = 9), but their applications are used in a broad spectrum of surgical specialties (Table 2; Figure 4).

ML algorithms have been used in a variety of surgical tasks, including assessment of basic surgical skills, such as suturing and knot tying (*n* = 12); basic laparoscopic (*n* = 13) and endoscopic training (*n* = 8); robotic-assisted surgical training (*n* = 13); and surgical simulation training (*n* = 8) (Table 2). According to our findings, the primary use of the technologies is for educational purposes.

## 4 Discussion

### 4.1 AI and ML

With the advent of AI, the aim was to design a system that could operate intelligently and would be able to understand human language, perform mechanical tasks, solve complex computer-based problems, and return a human-like answer. Another skill humans possess is the ability to learn from our environment, as in language acquisition. When children are exposed to language and start to recognize patterns, they learn the rules of the language. They practice, evaluate their progress, receive feedback, and make adjustments. ML is a subset of AI that allows an AI system to accomplish a similar feat (Figure 3). The capacity of the system to learn is based on 3 factors: the data consumed, quantification of how incorrect the current behavior is compared to the ideal or model behavior, and a feedback mechanism to help guide and produce better future behavior (86). ML technology can predict stock market trends and serve as the foundation for self-driving cars, and its potential applications in medicine are equally, if not more, transformative. ML has been demonstrated to be effective in disease prediction (13), disease detection (14), radiologic interpretation (15), and enhancement of surgical precision (16), the last of which is the focus of this article.

One of the most important aspects of an ML system is its algorithms. The algorithm is effectively the rule book that determines how the data will be used. A single algorithm could have hundreds or even thousands of “rules” or parameters defining its decision-making. Some examples of the algorithms that are used in surgical applications include the following:

• Convolutional neural networks (CNNs) (87)
• Support vector machines (88)
• Random forests (89)
• K-nearest neighbors (90)
• Recurrent neural networks and long short-term memory (LSTM) networks (91)
• Clustering algorithms (92)

These algorithms can be used in distinct learning paradigms, including supervised learning (93), unsupervised learning (86), and reinforcement learning (94). These 3 paradigms are the primary learning paradigms of ML, whereas deep learning is a distinct concept suitable for handling complex data structures (95).

#### 4.1.1 Supervised learning

Supervised learning is a methodology that trains AI by using a dataset that is already labeled. This would be the equivalent of giving a child a stack of images in which some are apples and others are bananas. If the images are labeled, the child can learn which label is associated with the apple and which with the banana. When a new image appears without a label, they can accurately label it.

Supervised learning deals with 2 problems: classification and regression. Regression problems involve predicting a continuous output with a value that can exist at any point within a range; an example would be predicting Medical College Admission Test scores based on hours studied. The classification problems ask the system to indicate a categorical output, such as benign or malignant (93). These systems are essential for problems that require accurate predictions and the ability to generalize new data. This learning can recognize and track instruments, hand movements, and anatomical landmarks in a surgical application.

##### 4.1.1.1 Application in using surgeon hand motions to predict surgical maneuvers

Azari et al. (33) asked 37 clinicians, ranging from medical students to retirees, to perform simple and running subcuticular suturing. The hand movements were recorded during the suturing tasks, and 3 ML techniques were applied: decision trees, random forests, and hidden Markov models. These techniques were used to classify surgical maneuvers every 2 s (60 frames) of video. The random forest predictions correctly classified 74% of all video segments, whereas the hidden Markov model improved this to 79%. The results highlight the potential of ML for predicting surgical maneuvers using video data. This approach is promising for improving surgical training and objective assessment by providing a more efficient way to review surgical videos.

#### 4.1.2 Unsupervised learning

Supervised learning requires data to be prelabeled, whereas unsupervised learning does not rely on any predefined labels for the data. Instead, it identifies intrinsic patterns within the data provided to the program. There are 2 different types of unsupervised learning: clustering and dimensionality reduction. Clustering involves the use of algorithms such as k-means and hierarchical clustering to group things together. Dimensionality reduction involves principal component analysis and T-distributed stochastic neighbor embedding, which are used to reduce the complexity of the data (86).

##### 4.1.2.1 Application in vision-based tracking of surgical instruments

Ryu et al. (2) demonstrated that they could identify the movement of surgical instruments and detect anomalies in their movements using an unsupervised learning method with k-means clustering. By combining this technique with a supervised CNN, they developed a collision warning system that enhanced surgical safety by reducing the risk of tissue perforation and instrument collisions.

#### 4.1.3 Reinforcement learning

Reinforcement learning is a type of ML that resembles unsupervised learning in that it does not use prelabeled samples for training. However, it interacts continuously with the environment, which provides feedback, and through this mechanism, it can produce a desired behavior (86). This type of learning focuses on optimization through trial and error, which makes it suitable for dynamic and complex environments like surgery. There is high potential for its use, with possibilities ranging from skill acquisition for surgical robots to real-time decision support and personalized surgical assistance.

#### 4.1.4 Deep learning

Deep learning is another subset of ML that relies on artificial neural networks with multiple layers. This form of ML excels at learning from large amounts of data, which makes it suitable for applications like healthcare, visual recognition, text analytics, and cybersecurity (96). Within deep learning, there are techniques that can be classified as supervised learning. These include CNNs and recurrent neural networks. CNNs are helpful for image recognition and analysis, whereas recurrent neural networks are suitable for sequential data like time series or natural language processing.

##### 4.1.4.1 Application in estimation of the 6-degree-of-freedom poses of surgical instruments

CNNs were used by Burton et al. (69) to estimate the 6-degree-of-freedom poses of surgical instruments. The CNN was trained on a large dataset of simulated and real-world images. In simulated scenes, CNN achieved mean in-place translation errors of 13 mm and mean long-axis orientation errors of 5°. These errors increased to 29 mm and 8° in the real-world scenes. This demonstrated that the CNN can effectively estimate the 6-degree-of-freedom poses of surgical instruments in real-time, offering an alternative to marker-based tracking methods.

##### 4.1.4.2 Application in hand tracking for surgical telestration

A study by Müller et al. (63) aimed to develop a real-time system that could track hand movements to use in surgical telestration, potentially aiding in surgical training via the augmented reality visualization of surgeons' hands. A total of 14,000 annotated images were used as the dataset, and the model architecture included bounding box detection, skeleton tracking, and hand segmentation. The system achieved a mean detection accuracy of 98% and a Dice similarity coefficient for hand segmentation of 0.95, indicating high segmentation accuracy. Deep learning proved effective for developing real-time hand-tracking systems.

##### 4.1.4.3 Application in neuronavigation

Kögl et al. (8) used this ML methodology to develop a tool-free neuronavigation system using a deep learning model, a single red-green-blue (RGB) camera, and a laptop. This system achieved a high level of accuracy with a target registration error of 1.3 cm and provided real-time guidance for craniotomy planning and execution.

##### 4.1.4.4 Application in hand gesture recognition

Karrenbach et al. (97) created a dynamic hand gesture recognition system using electromyography (EMG) signals and a deep learning methodology known as bidirectional LSTM. This system works by classifying hand gestures and reached an accuracy of 79.5% through a recalibration training scheme. This study highlighted the potential of this type of ML to improve the accuracy and reliability of hand gesture recognition systems.

##### 4.1.4.5 Application in instrument tracking for ophthalmic surgical maneuvers

Tang et al. (39) initially developed a deep learning–based adaptively sampled spectrally encoded coherence tomography and reflectometry (SECTR) program for intraoperative optical coherence tomography for real-time imaging during ophthalmic surgical maneuvers. A deep learning model with a CNN was used to track surgical instrument positions and sample instrument tips. The study presented a novel real-time automated tracking of surgical instruments using deep learning.

Tang et al. (3) used a similar method to track surgical instruments, and the results demonstrated a resolution-limited tracking accuracy with a mean pixel error statistically significantly lower than the resolution limit of 2.95 pixels. This finding was also true for speed, depth, and orientation variations. This technology combines multimodal imaging with deep learning and shows high accuracy and speed. It shows promise for improving surgical outcomes by enhancing surgical visualization. Furthermore, tracking surgical instruments can help achieve greater precision during procedures, reduce errors, and make surgical interventions more effective.

##### 4.1.4.6 Application in automating hand tracking using computer vision

Zhang et al. (4) also developed a method to automate the detection and tracking of surgeon hand movements using computer vision and a CNN. Data were collected from publicly available YouTube videos annotated with spatial bound boxes. The results showed an average mean precision of 70.4%, and 75.4% with fine-tuning of the dataset. The findings highlight the potential of computer vision for effective hand tracking. It provided a foundation for developing automated systems for surgical skill assessment, leading to improved surgical outcomes and training.

##### 4.1.4.7 Application in video-based surgical skill assessment using three-dimensional (3D) CNNs

Funke et al. (37) investigated a method for automatic, objective assessment of surgical skills using only video data. Their study, which used a 3D CNN and a temporal segment network trained on a kinetics dataset, demonstrated significant performance improvements. The approach achieved high classification accuracies for surgical skill assessment, with accuracy ranging from 95.1% to 100%. The findings reaffirm the reliability of the deep learning approach in evaluating surgical skills, paving the way for advancements in surgical training and outcomes.

##### 4.1.4.8 Application in instrument segmentation with TernausNet during temporal bone surgery

Yuan et al. (9) used an ML application known as TernausNet, a deep learning method, to achieve accurate segmentation of surgical instruments during temporal bone surgery. Video footage of 15 cochlear implant surgeries was collected, with a total of 744 annotated images. Results were measured as F1 dice scores, a single metric that combines precision and recall, evaluating the accuracy of a model. Binary segmentation achieved an F1 dice score of 95% during training and 87% during validation, and multiclass segmentation achieved an F1 score of 62% during training and 61% during validation. Intersection over union for binary segmentation was 91% during training and 77% during validation. Overall, the findings confirm the accuracy and practicality of ML-based segmentation.

### 4.2 Types of sensors used in surgical motion and hand tracking

Technological advancements have enabled the development of hand-tracking tools to effectively analyze the motion of surgical instruments and hands, which can be used to train surgical residents and enhance their technique and motion efficiency. Several sensor systems for tracking surgical motions have been proposed and used, including optical, inertial, electromagnetic, ultrasonic, and hybrid systems that combine different tracking systems (Table 2). Here, we summarize the different types of sensors, their functions, and their applications in tracking surgical hand motions.

#### 4.2.1 Vision-based methods

**Figure 5:**
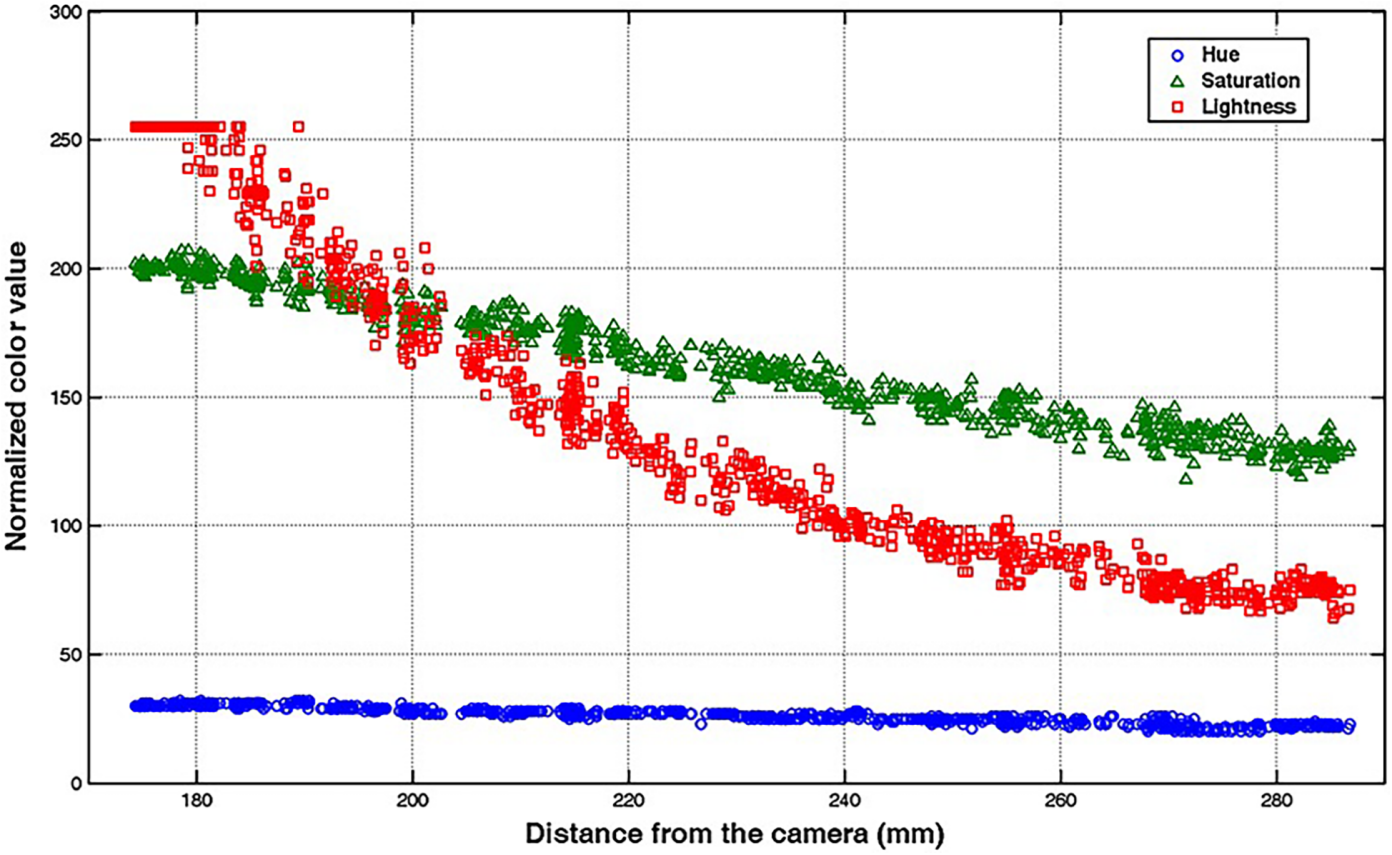
Vision-based method for surgical-instrument tracking. Loukas et al. (98) have proposed an adaptive color update for the endoscopic instrument-tracking process. This figure depicts the mean color values, including hue, saturation, and lightness, in relation to the instrument's movement away from the endoscope's camera. The marker's color significantly changes as the instrument moves in the training box. *Used with permission from Loukas C, Lahanas V, Georgiou E. An integrated approach to endoscopic instrument tracking for augmented reality applications in surgical simulation training. Int J Med Robot. 2013;9*(*4*)*:e34-51*.

Early techniques for tracking surgical instruments typically relied on vision-based methods. One example is a 2014 study by Partridge et al. (22), where colored bands on surgical instruments were used for motion tracking via computer vision processing. Similarly, a 2013 study by Loukas et al. (98) combined vision-based tracking using colored tips with the Hough-Kalman approach. Adaptive markers, capable of dynamically changing colors as the surgical instrument moved within the training box, were used (Figure 5) (98). More recently, computer vision processing has also been used to recognize surgical instruments to prevent surgical objects from being left behind during operations (99). Since then, newer and more precise methods of tracking instruments and hand motions have been proposed.

#### 4.2.2 Optical sensors

Optical sensors, such as the Polaris Vega and Stryker NAV3i systems, use infrared light and navigation markers to precisely track surgical motions. A fixed reference tool near the surgical field aids in localizing movements within the surgical volume, as the optical tracker detects infrared light reflected from the markers to determine the coordinates of the labeled instrument (100). Challenges like visual occlusion have been addressed by implementing multicamera modules, enabling instrument tracking from multiple angles (100).

#### 4.2.3 Simple video recordings

Simple video recordings of surgeries can also be used to effectively analyze and monitor the movement of surgical instruments and hands during operations. For example, motion-tracking systems can assign coordinates to instruments in selected videos, depicting the same surgical procedure with varying depth, surgical side, magnification, and resolution (10, 83). These datasets can be stored in various formats and analyzed using CNNs to enable precise motion tracking. In a study by Raymond et al., mastoidectomy surgical videos were used to track surgical instruments (83). The datasets were stored in COCO JSON format, and Detectron, an open-source Facebook AI research CNN, was employed to identify the surgical instruments. Subsequently, the motions of these instruments were tracked using the mastoidectomy instrument tracking module (83).

#### 4.2.4 Automated instrument tracking

**Figure 6:**
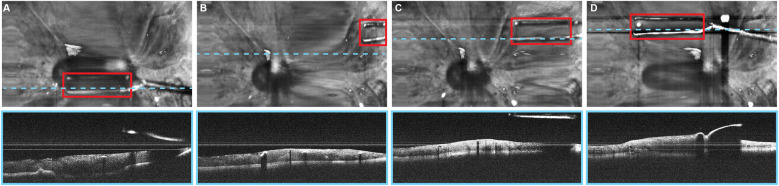
Automated instrument-tracking using SECTR. Tang et al. (39) trained a CNN to track 25G ILM forceps, a surgical instrument, to allow 4D visualization of the surgical motion during ophthalmologic surgeries. **(A)** Five-averaged SER image and representative OCT image demonstrating the tip of the forceps (*red box*) in an ex vivo bovine eye. Movement of the instrument out of the OCT plane (*blue dashed line*) and adaptive sampling can be seen on **(B–D)**. CNN, convolutional neural network; OCT, optic coherence tomography; SECTR, spectrally encoded coherence tomography and reflectometry; SER, spectrally encoded reflectometry; 4D, four-dimensional. *Used with permission from Tang E, El-Haddad M, Malone JD,* et al. *Automated instrument-tracking using deep-learning-based adaptively-sampled spectrally encoded coherence tomography and reflectometry (SECTR). Investigative Ophthalmology & Visual Science. 2019;60(9):1276-1276*.

Automated instrument tracking using SECTR has also been proposed. This method was used in 2 studies by Tang et al. (39, 47), in which the movements of the 25G ILM forceps, a surgical instrument, were tracked by spectrally encoded reflectometry. The forceps were tracked by training a CNN, allowing 4-dimensional (4D) visualization of surgical motion during ophthalmological surgeries (Figure 6) (39, 47).

#### 4.2.5 Tool-free methods

Tool-free methods of tracking hand motion have been explored. In a study conducted by Kögl et al., a single RGB camera combined with ML-based hand pose estimation was used to track hand motion (8). The model was trained using 21 hand landmarks with both real and synthetic images. The Perspective-*n*-Point problem was then solved, and 3D coordinates of the hand landmarks were obtained. Although the study aimed to facilitate tool-free neuronavigation by using a fingertip to select anatomical landmarks, it faced challenges caused by a high level of interference due to 3D landmark positions. To address this, 2 methods were implemented to filter out the noise.

**Figure 7:**
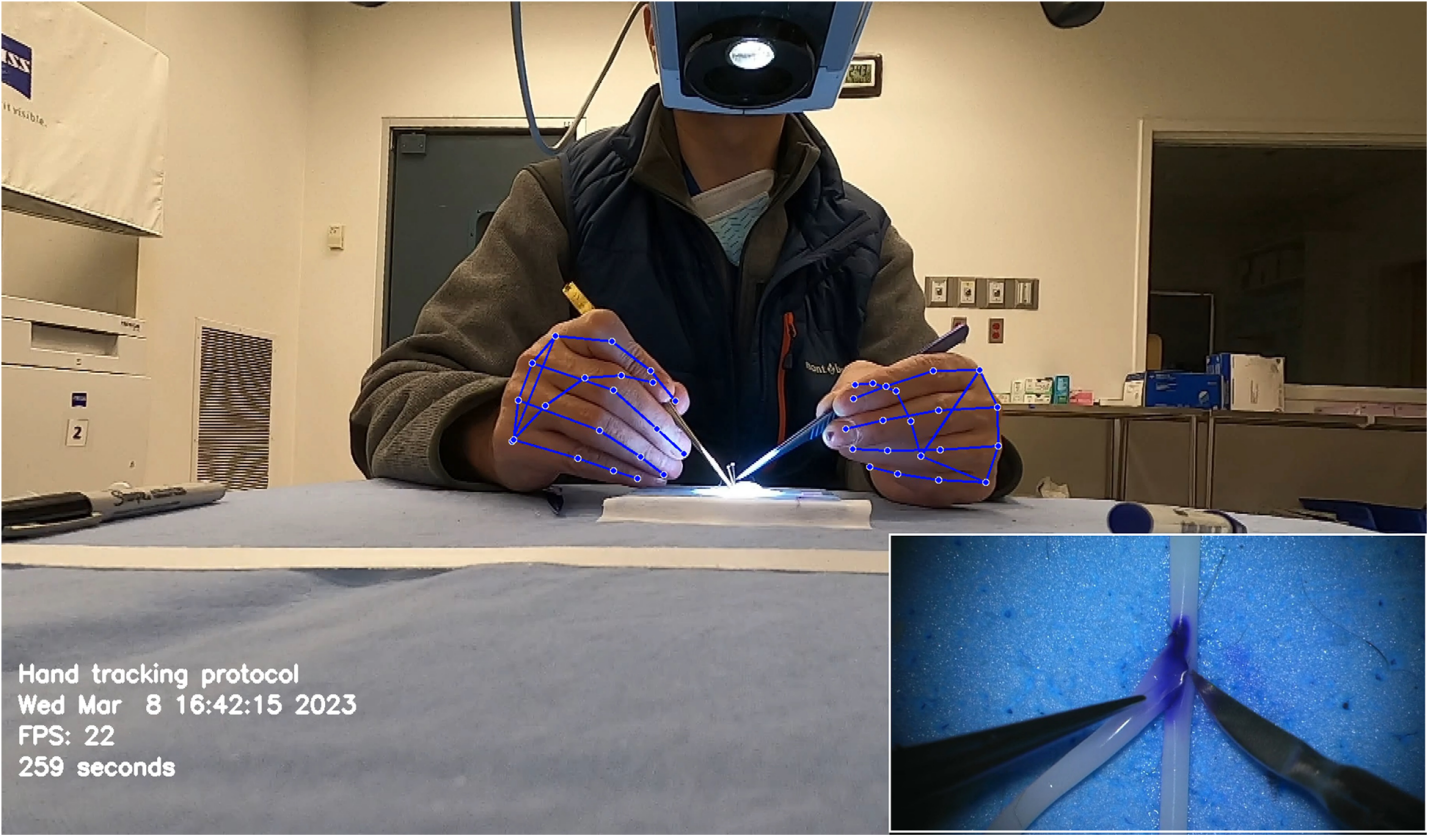
Hand tracking using a machine learning system developed by Google (Mediapipe) (https://ai.google.dev/edge/mediapipe/solutions/guide). The neural network automatically detects hands without requiring preliminary registration. The hand-tracking process starts with activating a palm detector, followed by a landmark detector once the hands are located in the image. At the beginning of the hand-tracking session, the operator is asked to display both hands to the camera to ensure swift activation of the model. The effectiveness of hand detection is validated by displaying real-time tracking data during the simulation. This photograph was taken during microvascular anastomosis training at the Loyal and Edith Davis Neurosurgical Research Laboratory, Barrow Neurological Institute, in Phoenix, Arizona. *Used with permission from Barrow Neurological Institute, Phoenix, Arizona*.

Similarly, Gonzalez-Romo et al. (11) reported a sensor-free hand-tracking method using a motion-detector program created with the Python programming language (Python Software Foundation, https://www.python.org/) and a machine learning system developed by Google (Mediapipe) (https://ai.google.dev/edge/mediapipe/solutions/guide). The aim was to analyze hand movements during microanastomosis training. They also demonstrated the effectiveness of ML in assessing hand movement metrics without any physical sensors (Figure 7) (11).

#### 4.2.6 Surgical instrument tracking and hand-eye coordination

**Figure 8:**
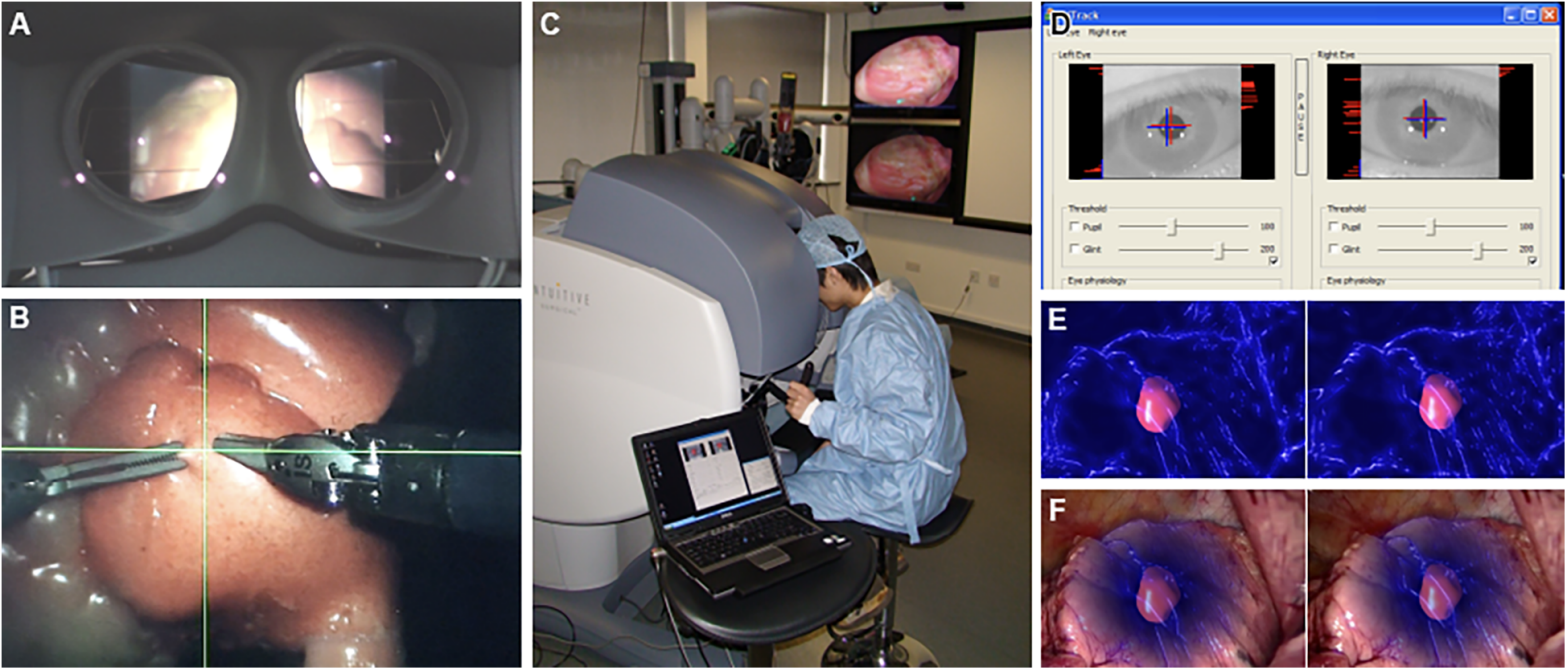
Gaze-contingent perceptual docking. Yang et al. (101) presented the general concept of perceptual docking for robotic control. The aim was to investigate the potential use of perceptual docking for knowledge acquisition in robotic-assisted minimally invasive surgery. This figure represents the different aspects of the gaze-contingent perceptual docking. **(A)** The viewpoint of the surgeon using this system. **(B)** The surgical field and surgical instruments are shown intraoperatively. **(C)** External view of the surgeon and the system. **(D)** The binocular tracking of the eyes. **(E and F)** The application of augmented reality to gaze-contingent eye tracking, showing **(E)** nonphotorealistic augmented reality rendering and **(F)** fused with the original video. *Used with permission from Yang G-Z, Mylonas GP, Kwok K-W,* et al. *Perceptual Docking for Robotic Control, in: T. Dohi, I. Sakuma and H. Liao (Eds.), Medical Imaging and Augmented Reality, Berlin, Heidelberg: Springer Berlin Heidelberg. 2008;21–30*.

Systems like the one proposed by AbuSneineh and Seales (20) measure hand motions along with heart rate and body movements, including those of the arms, head, and eyes, using dedicated cameras (20). Yang et al. explored hand-eye coordination, hypothesizing that a gaze-contingent framework using binocular eye-tracking could aid robotic surgeries (Figure 8) (101). The results highlighted that enhanced surgical instrument manipulation and hand-eye coordination could be achieved by analyzing surgeons’ saccadic eye movements and ocular vergence (101).

#### 4.2.7 Inertial sensors

**Figure 9:**
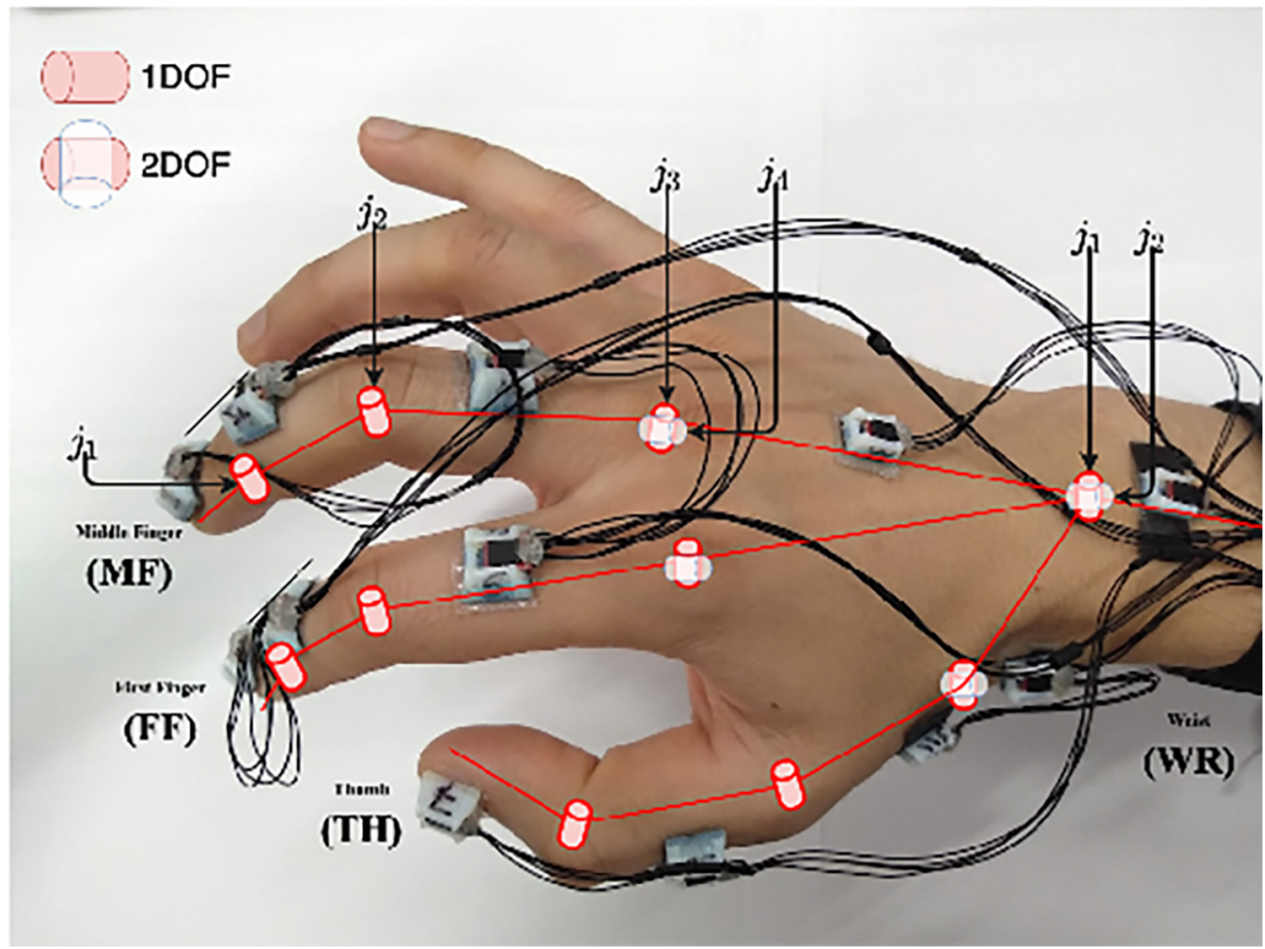
Inertial sensors. Sani et al. (5) have used inertial sensors for recording and mapping a surgeon's hand's gross and fine motions during cardiac microsurgery. This figure is a pictographic representation of the 12-unit-IMU sensor device used in this study. Each sensor is attached to a specific joint to allow precise measurement of position change. The DIP and PIP joints are represented by *j*_*1*_ and *j_2_*, respectively. The axes of the MCP joint are represented by *j_3_* and *j_4_*. DIP, distal interphalangeal joint; IMU, inertial measurement unit; MCP, metacarpophalangeal joint; PIP, proximal interphalangeal joint. *Used with permission from Sani MF, Ascione R, Dogramadzi S. Mapping Surgeon's Hand/Finger Motion During Conventional Microsurgery to Enhance Intuitive Surgical Robot Teleoperation. ArXiv. 2021;abs/2102.10585*.

Inertial sensors track motion based on the principle of inertia, which states that an object continues to move unless acted upon by an external force. These wearable sensors include accelerometers (tracking linear acceleration), gyroscopes (measuring angular velocity), and magnetometers (detecting magnetic field changes) (102). These different components are typically integrated into an inertial measurement unit (IMU) when used to detect human motion and posture to increase the accuracy of their measurements (102). The application of inertial sensors has been shown to be successful, with Sani et al. using a chain of 12 IMUs for surgical hand tracking (Figure 9) (5). These wearable IMUs focus on accurately tracking 4 main motions: joint angles of the digits and wrist, global hand position and orientation, global instrument position and orientation, and jaw angle of surgical instruments. The goal was to increase the degree of freedom to allow for smoother and more natural movements by the surgeon (5). Given that the index, middle finger, and thumb are the significant contributors to fine grasping and the use of the surgical instruments (in this case, Castroviejo needle-holder and forceps), each finger was tracked by 3 separate IMUs. The wrist joint was tracked via 2 IMUs, a reference point IMU on the forearm and one on the palm, with the rest of the IMUs being placed strategically to capture the best movements of the remaining joints of the hand.

The Polaris Spectra (Northern Digital Inc.) optical sensors were used to precisely track global hand position and orientation, with infrared markers attached directly to the Castroviejo surgical instruments and the surgeon's wrist (5). A significant disadvantage of the IMU system, however, is the accumulation of small errors in position estimation over time, leading to a reduced accuracy of tracking. These sensors typically drift over time and are sensitive to surrounding magnetic interference. To mitigate this issue, this study attempted to decrease drift by measuring relative angles between IMUs; in addition, they recalibrated the recordings every 5 min (5). IMU sensors have also been combined with optical tracking methods to overcome the line-of-site barrier associated with optical tracking methods (103).

#### 4.2.8 Multimodal sensor systems

Multimodal systems integrate different sensor types, such as inertial and EMG technologies, to enhance tracking accuracy and address the limitations of single-sensor modalities. Kowalewski et al. published a study in which 28 participants, including 8 novices, 10 intermediates, and 10 experts, completed laparoscopic suturing and knot tying while wearing the Myo armband, a motion-sensing EMG technology combined with inertial sensors that can interpret and track motion (36). The inertial sensors within this technology can measure angular velocity, acceleration, and arm orientation in addition to Euler orientation (36).

EMG electrodes, available as dry or wet types, have been used to track hand motion (97). Dry electrodes are easy to use and attach directly to the skin, whereas wet electrodes require gel or paste but provide higher-quality electrical signals. These EMG electrodes come in both a wireless and a wired form. In a proof-of-concept study by Karrenbach et al. (97), hand gesture recognition using EMG electrodes was demonstrated using the MiSDIREKt (Multisession Single-subject Dynamic Interaction Recordings of EMG and Kinematics) dataset. This EMG hardware consisted of an armband with a wireless 8-channel surface EMG using dry electrodes made of carbon-black infused thermoplastic polyurethane. It tracked various hand gestures, motions, and dynamic interactions (97).

#### 4.2.9 Electromagnetic sensors

Electromagnetic sensors measure the voltage change created by object movement in space. In the case of hand-tracking models, a coil is wrapped around a ring on a finger (104). This coil is fitted with a printed circuit board that generates a current through the coil, creating a magnetic field signal. This magnetic field signal is then detected by sensors attached to a wristband, which approximate the coil's pose on the finger. However, this is not an exact measurement; a calibration process is needed to help filter out parameters such as noise, gain, and bias. These systems use magnetic fields to track object movement without line-of-sight limitations, but their accuracy can be affected by device interference and surrounding magnetic noise (104). A study by Bordin et al. utilizing an augmented reality helmet, HoloLens 2, found promising results when applying this technology to hand tracking (105). This study used an electromagnetic measuring system to track hand motion (categorized as either no hand motion, simple hand motion, or hand motion while using a 3D-printed object) by wearing linked sensors on the index finger and thumb while wearing the HoloLens 2 helmet (105).

#### 4.2.10 da Vinci Surgical System

**Figure 10:**
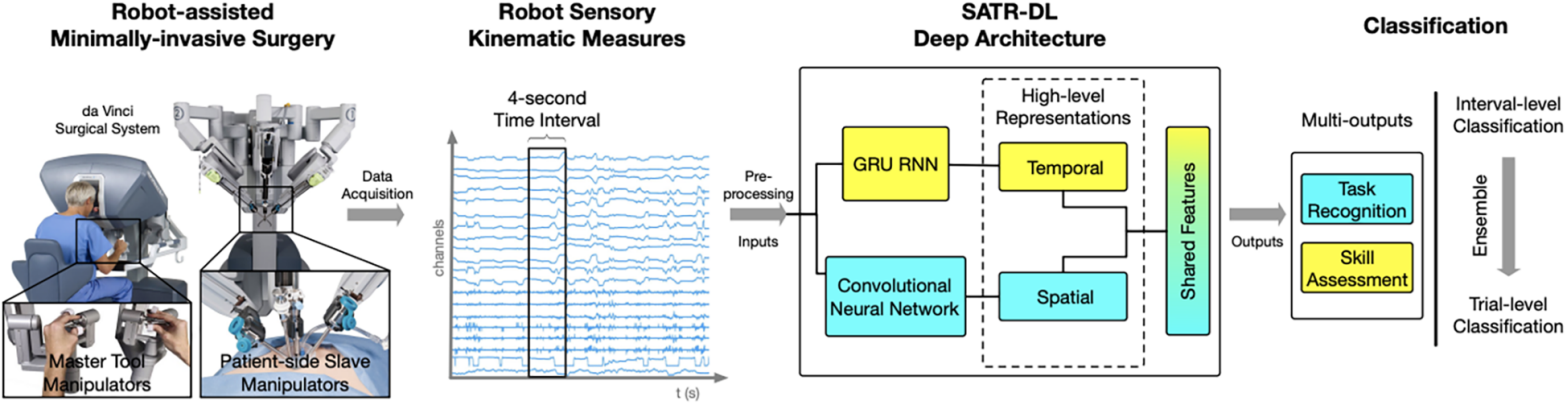
Surgical skill assessment and task recognition. Wang and Fey (30) presented an analytic framework with a parallel deep learning architecture, SATR-DL, to evaluate trainee performance and to recognize surgical training activity in robotic-assisted surgery. They aimed to improve surgical skill assessment and task recognition with deep neural networks. This figure illustrates a complete framework stated in this study, flowing from left to right. The far left image shows the da Vinci Surgical System, with which a 4-second clip of the surgical motion is captured using da Vinci end effectors. These data are then captured and converted to kinematic measurements, which are then analyzed by the SATR-DL deep architecture. Once analyzed, the measurements are classified into task recognition and skill assessment. GRU, gated recurrent unit; RNN, recurrent neural network; SATR-DL, skill assessment and task recognition with deep learning. *Used with permission from Wang Z, Fey AM. SATR-DL: Improving Surgical Skill Assessment And Task Recognition In Robot-Assisted Surgery With Deep Neural Networks, 2018 40th Annual International Conference of the IEEE Engineering in Medicine and Biology Society (EMBC). 2018;1793–1796*.

Hand motion has also been tracked using advanced systems like the da Vinci Surgical System. Wang and Fey used an end-to-end motion analysis framework with a multioutput deep learning architecture (SATR-DL) to track surgical trainee motions as raw kinematics during specific tasks (30). This method allowed for the representation of both spatial and temporal dynamics of hand motion during surgical tasks. Once the raw motion kinematics had been recorded, they could be input into SATR-DL to recognize the specific task and assess skill level (Figure 10) (30).

### 4.3 Applications of ML and hand and instrument tracking in surgery

ML and hand-tracking technologies are rapidly advancing and can potentially improve a range of healthcare technologies in areas such as preoperative planning, teaching modalities, intraoperative assistance, postoperative analysis, and rehabilitation. This section discusses recent and upcoming developments in ML and hand-tracking technologies and the potential for beneficial disruption in the surgical field. Some limitations of these technologies are addressed, and the developments necessary to ensure their widespread integration and use in the surgical field are discussed.

#### 4.3.1 Surgical instrument tracking

One of the most significant opportunities for ML and hand-tracking technologies to disrupt the surgical field is with intraoperative assistance. One way these technologies can be used is by using computer models to track and record the movement of surgical instruments in the hands of different surgeons.

Zhang and Gao proposed a marker-free surgical instrument tracking framework based on object extraction via deep learning (49). They compared their deep learning framework with a marker-free tracking-by-detection framework. They found that the proposed deep learning framework improved accuracy by 37%, compared with a 23% improvement using the other model. The findings highlight the rapid development of accurate and efficient tracking technologies for use in various procedures (49).

Surgical instrument tracking has also been used with analysis by AI. Baldi et al. created a model of an eye and then recorded several videos in which surgical instruments were moved throughout the model eye. Several characteristics of the surgical tools were labeled in the frames of the videos, such as location, depth, and tool type (6). Two different deep learning models were trained to predict the tool characteristics, and their performances were recorded. They found that the accuracy of the classification model on the validation dataset was 83% for the x-y region, 96% for depth, 100% for instrument type, and 100% for laterality of insertion. The close-up detection model was performed at 67 frames per second with a mean average precision of 79.3%. The findings illustrate how deep learning models can enable software-based safety feedback mechanisms during surgery and document metrics to guide further research for optimizing surgical outcomes (6).

#### 4.3.2 Objective skill assessment and task recognition in surgery

Objective skill assessment and task recognition in surgery have also been areas of interest in using deep neural networks. Wang and Fey created an analytic framework with a parallel deep learning architecture, SATR-DL, to assess trainee expertise and recognize surgical training activity (30). According to the results, the deep learning model was able to successfully recognize trainee skills and surgical tasks, including suturing, needle passing, and knot tying. The reported accuracies of the deep learning model were 0.960 and 1.000 in skill assessment and task recognition, respectively. Ultimately, the findings underscored the potential of these neural networks for efficient quantitative assessments in surgical applications, including robotic surgery (30).

#### 4.3.3 Pose estimation of the surgical tool

Sani et al. attempted to develop a system to train a deep neural network by mapping real-time data acquisition inputs of wrist, hand, and surgical tools during mock-up heart microsurgery procedures (5). The proposed network was trained to estimate the poses of the tools from refined hand joint angles, and the outputs were surgical tool orientation and jaw angle obtained by an optical motion capture system. It was found that the neural network could accurately estimate the surgical instrument's pose from the recorded hand movements of the surgeon, achieving a mean squared error of less than 0.3%. This system is suggested to potentially improve the remote teleoperation of similar surgical tools in microsurgery (5).

#### 4.3.4 Applications in robotic surgery

Deep learning models can also potentially be used to evaluate surgical skills in robotic surgery procedures. One of the difficulties with assessing these skills is that quantifying them requires having an accurate way to track the movement of surgical instruments (7). Lee et al. proposed a deep learning–based surgical instrument tracking algorithm to evaluate surgeons' skills in performing robotic surgery procedures. They overcame the drawbacks of occlusion and maintenance of the identity of the surgical instruments, and motion metrics were used to develop the deep learning models (7). It was found that the surgical skill prediction models had an accuracy of 83% with Objective Structured Assessment of Technical Skill and Global Evaluative Assessment of Robotic Surgery. Ultimately, it was argued that the performance of the deep learning model demonstrated how an automatic and quantitative method could be used to evaluate surgical skills, in contrast to previous methods (7).

#### 4.3.5 Endoscopic instrument tracking

Loukas et al. also adequately addressed the issue of occlusion handling by using 2 different tracking algorithms for endoscopic instrument tracking (98). The proposed method was validated based on several image sequences for tracking efficiency, pose estimation accuracy, and applicability in augmented reality-based training. The absolute average error of the tip position for the tool was 2.5 mm, and the average error of the instrument's angle to the camera plane was 2°. The approach showcased promising results for applying augmented reality technologies to laparoscopic skill training using a computer vision framework (98).

#### 4.3.6 Intraoperative real-time feedback

Although ML and hand-tracking technologies have been documented to analyze surgical techniques retrospectively, there has also been a significant interest in developing models that can provide real-time feedback in intraoperative settings. Yibulayimu et al. used ML to create a model that automatically evaluated the trainee's performance of liposuction surgery and provided visual postoperative and real-time feedback (67). The model was assessed on liposuction surgery datasets, with an optimistic accuracy ranging from 89% to 94%. The findings highlight the potential for ML and other technologies to provide immediate feedback to surgical professionals (67).

#### 4.3.7 Surgical skill assessment during cataract surgery

Morita et al. sought to use the potential of ML in evaluating the surgical skills of ophthalmologists in cataract surgery (45). They proposed a method that used image classification and segmentation networks to detect surgical problems and the surgical instrument, respectively. The network could detect surgical problems with an area under the receiver operating characteristic curve of 0.97 and detect the tips of the forceps and incisional sites with 86.9% and 94.9% detection rates, respectively (45).

#### 4.3.8 Instrument tracking and adaptive sampling models for 4D imaging

Tang et al. proposed a deep learning–based automated instrument tracking and adaptive sampling model that could be used for 4D imaging of ophthalmic surgical maneuvers (3). The findings indicate that real-time localization of surgical instruments can be achieved using SECTR with the deep learning model. Moreover, adaptive sampling enhances the visualization of instrument-tissue interactions by increasing sampling density without sacrificing speed (3).

#### 4.3.9 ML-based surgical workflow analysis

Using surgical videos for AI-based analysis holds immense potential to identify defects and refine surgical techniques. Khan et al. (106) demonstrated this potential by employing Touch Surgery (Digital Surgery Ltd.) to develop and validate an ML-driven stage and procedure analysis tool. Expert surgeons labeled the stages and steps of 50 anonymized endoscopic transsphenoidal surgery videos, with 40 videos used to train a CNN. Based on 10 repetitive videos by Touch Surgery, the model's evaluation showed promising results, with the ML model's automatic detection of surgeons' stage and procedure recognition.

The results indicated that the longest phase was the sellar phase (median 28 min), followed by the nasal phase (median 22 min) and the closing phase (median 14 min). The longest step was tumor identification and resection (median 17 min). In comparison, posterior septectomy and sphenoid septation resection (median 14 min) and anterior sellar wall (median 10 min) varied considerably within the recorded stages.

Despite variations in video appearance, stage duration, and stage sequence, the model demonstrated high accuracy in recognizing surgical steps (91% accuracy, 90% F1 score) and procedures (76% accuracy, 75% F1 score). These findings underscored the reliability of ML-based surgical workflow analysis, which has numerous potential applications, such as education, operational efficiency, and patient outcomes (106).

#### 4.3.10 AI-based surgical phase recognition and analysis

Cheng et al. (107) presented an AI-based automated surgical phase recognition system in laparoscopic cholecystectomy. That study represented an excellent attempt to combine the capabilities of CNNs and LSTM networks to recognize the operative phases of a sequence of frames from a given laparoscopic cholecystectomy video. Although it is well-known that CNNs and LSTMs are highly effective techniques for analyzing medical data, this work leveraged CNNs to extract spatial information from adjacent pixels to generate frame-based visual features. Then, it passed them to an LSTM to capture the temporal transitions of the visual features across consecutive frames. This approach achieved very high accuracy in laparoscopic cholecystectomy operative phase recognition.

Kitaguchi et al. (108) aimed to develop a deep learning model for automatic surgical phase recognition during video laparoscopic sigmoidectomy. This model can be used for real-time phase recognition and to evaluate the accuracy of recognizing surgical steps and actions using image data via a CNN. The automatic surgical phase recognition achieved an accuracy of 91.9%, whereas the recognition of extracorporeal actions and irrigation reached accuracies of 89.4% and 82.5%, respectively. Additionally, the system performed real-time phase recognition at 32 frames per second, with the CNN facilitating the recognition of surgical steps and actions based on manually annotated data. The results highlight the system's high accuracy in automatically recognizing surgical steps and specific actions. Furthermore, it confirmed the feasibility of a real-time automatic surgical phase recognition system with a high frame rate (108).

#### 4.3.11 Future directions

Ultimately, expanding ML and hand-tracking technologies pose considerable opportunities for disruption in the surgical field. From improving the ability of trainees to learn valuable techniques through retrospective analysis to real-time feedback in intraoperative settings, there are myriad applications for these technologies. One limitation of these technologies is the lack of more extensive validation studies to more comprehensively evaluate the accuracy of these ML and hand-tracking models. In addition, future research needs to be done across a more diverse set of procedures to showcase these technologies' potential for beneficial disruption. Nonetheless, these technologies are rapidly expanding and improving, and surgical professionals should strongly consider how they can improve various areas within the field.

### 4.4 Applications of ML and hand and instrument tracking in neurosurgery

**Figure 11:**
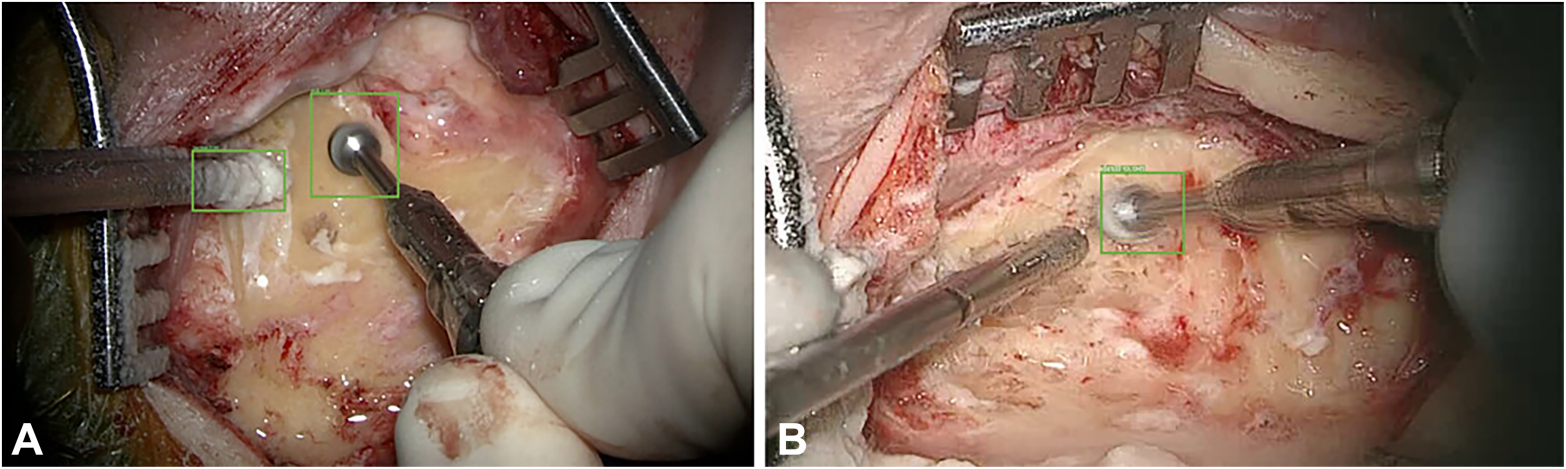
Machine learning (ML) applications in neurosurgery. Raymond et al. (83) tried to develop a convolutional neural network–based computer vision model to track 2 surgical instruments used in mastoidectomy procedures (drill and suction irrigator). Intraoperative video recordings were used to teach the model. Then, the model measured the outcomes, such as accuracy, precision, and average precision. The study stated 2 extremes regarding detection accuracy. **(A)** Detection accuracy for both the drill and the suction-irrigator was 100%. **(B)** Detection accuracy for the drill was 98%, but absent for the suction irrigator. Green bounding boxes were generated by the ML model to enhance visualization. *Used with permission from Raymond MJ, Biswal B, Pipaliya RM, Rowley MA, Meyer TA. Convolutional neural network-based deep learning engine for mastoidectomy instrument recognition and movement tracking. Otolaryngol Head Neck Surg. 2024;170(6):1555–60. doi: 10.1002/ohn.733*.

AI and ML have been applied to neurosurgery in several ways. Deep learning approaches have allowed neurosurgeons to track the movements of instruments intraoperatively. For example, in a paper by Raymond et al., a deep learning network was initially trained with images from surgery to recognize and track surgical instruments. The Residual Network 101, a CNN consisting of 101 deep layers, was used. These types of networks work by deconstructing aspects of the image and analyzing the different spectral bands in the image, allowing the network to identify specific components within the image. From here, the images were deconstructed in both low and high levels of resolution, eventually allowing for every detail of the image to be identified. This process is crucial for properly identifying surgical instruments within a video. After the deconstruction of these images, they were analyzed by a region proposal network, which allows for determining regions of interest. Once regions of interest have been identified, they are tested by the CNN to determine the accuracy of instrument recognition and prediction accuracy. Bounding boxes are used as a pictographic representation of these predictions. A computer vision model was used to track instruments during mastoidectomy surgeries. These deep learning technologies allowed the authors to track the exact coordinates of the surgical instruments during the procedure. Images from the procedure were analyzed as described above to determine the precise coordinates of these instruments and the bounding boxes. The model then measured outcomes such as the intersection over union ratio, accuracy, precision, and average precision (Figure 11) (83).

**Figure 12:**
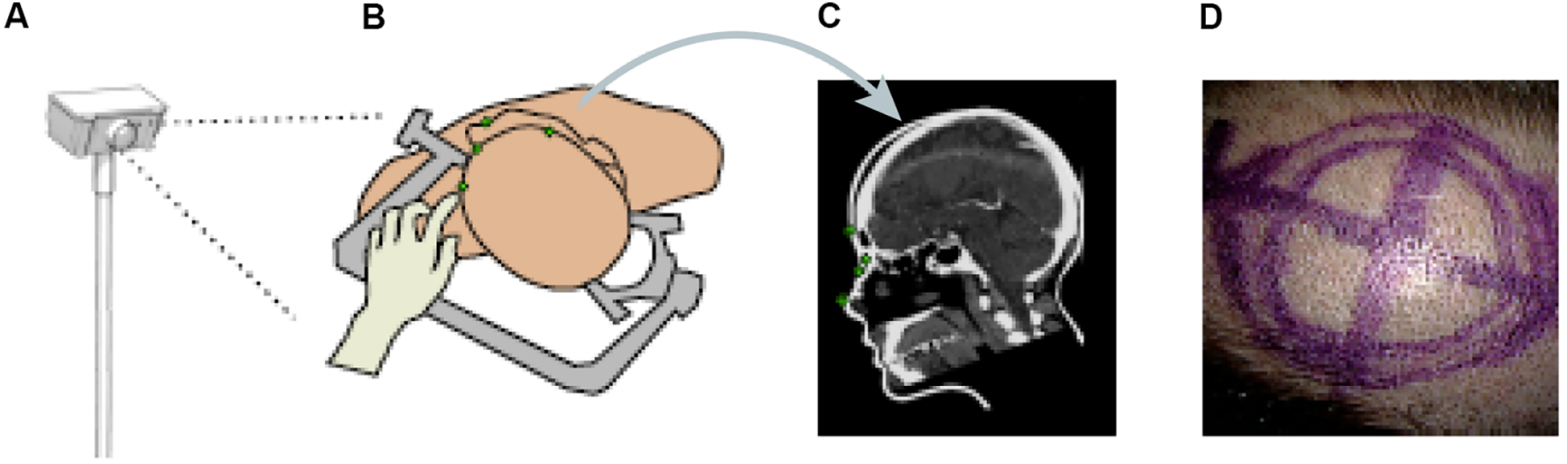
Machine learning (ML) application in neurosurgery tool-free methods. In an ML-based hand pose estimation study, Kögl et al. (8) presented a new tool-free neuronavigation method using an RGB camera during burr hole placement. This figure shows the stepwise process used to track hand motion for landmark selection. **(A)** Illustration of the monocular RGB camera used for this experiment to capture the motion of the hand. **(B)** Illustration of the hand being used to select landmarks for burr hole placement. **(C)** Computed tomography showing predefined anatomical landmarks. **(D)** Photograph showing the final position of the burr hole placement for a craniotomy using this system. *Used with permission from Kögl FV, Léger É, Haouchine N,* et al. A Tool-free Neuronavigation Method based on Single-view Hand Tracking. *Comput Methods Biomech Biomed Eng Imaging Vis*. 2023;11(4):1307–1315. doi:10.1080/21681163.2022.2163428.

Other papers have also proposed ML methods to track objects or hands' movements in surgery. Kögl et al. published a study in 2022 describing the use of ML to track the movements of hands via hand topology (8). This tool-free neuronavigational method focused on 21 landmarks assigned to a hand and used real and synthetic images to train 2 deep learning networks to estimate hand pose. Real images were used to understand the 2-dimensional coordinates of the hand, whereas synthetic images were used to train the model on relative depth. The Google MediaPipe ML framework was implemented to determine the 2.5-dimensional hand pose. However, the aim was to estimate the 3D pose. Therefore, once these images had been used to train the neural networks, solving the Perspective-n-Point problem was then necessary. This was done by determining where the hand was in space relative to the frame, allowing the determination of the 3D hand pose. Once the 3D hand pose had been estimated, these coordinates were inputted into the 3D Slicer using the OpenIGTLink. The overall aim was to have tool-free neuronavigation that would allow the estimation of hand pose to allow neurosurgeons to use their fingertips to plan craniotomies instead of conventional pointers (Figure 12) (8).

Similar methods of hand pose estimation have been used in neurosurgical procedures such as microanastomosis. Gonzalez-Romo et al. published a study that used 21 hand landmarks in conjunction with ML to identify gross and fine movements during microanastomosis procedures for evaluating surgeons' performance (Figure 7) (11). The Python programming language was used to develop a motion detector incorporating Google Mediapipe (https://ai.google.dev/edge/mediapipe/solutions/guide). This motion detector was able to analyze videos of anastomosis procedures to track the motion of hands in a physical sensor-free manner.

In a recent study by Kim et al. (82), a deep learning–based object detection model (YOLOv8 OBB Framework) was used to estimate the accurate localization of spinal fixation instruments during spinal surgery. The model was trained using various datasets of images from different manufacturers. Their model's F1 score is 0.86 (1.00 precision and 0.80 recall). Although the model had high precision, the study stated that it had some limitations in detecting all the instruments present.

Applications of ML algorithms in neurosurgery are on the rise and have a significant potential for development. As improvements in ML technologies progress, their integration into neurosurgery is becoming more widespread. This trend highlights the opportunities for leveraging ML algorithms to enhance neurosurgical procedures such as diagnosis, preoperative surgical planning, and outcome prediction.

### 4.5 Challenges, ethical considerations, and future directions

Despite the rapid advancement of ML and hand-tracking technologies in recent years, their implementation in clinical settings still faces several limitations. Technical challenges such as data quality and integration from various sensors, as well as real-time processing and computational limitations, are at the forefront of these limitations. However, the eventual expansion of platforms such as video acquisition can help address some of these computational concerns. Filicori et al. surveyed currently available video recording technology companies and found that those on the market already allow for in-depth performance analysis through manual and automated review (109). In addition, these devices have the potential to be integrated into future robotic surgical platforms.

From another perspective, the integration of AI algorithms into surgical practice may heavily rely on human effort. For instance, successful surgical instrument detection requires surgical videos to be annotated, and even frame by frame labeling or categorization may be necessary. Although this process is time-consuming, it is crucial for providing the necessary visualization to AI algorithms (110). The use of these models in surgical practice has not always been successful. Although these models have shown success in laparoscopic procedures, they have been less effective in open surgical procedures. This may be because of the complex anatomy encountered during open surgeries and the greater difficulty in annotating video frames of open surgical procedures compared to laparoscopic ones. More studies are needed to enhance the potential applications of AI in open surgical procedures (111).

Another concern may be the cost and infrastructure needs associated with integrating these technologies. However, Dayak and Cevik (12) proposed a computer vision–based simulator using a single camera and planar mirror that improved accuracy by 60% over relevant methods in the literature, even at low resolutions and low processing time. The authors also argued that their method was a low-cost alternative for computer vision–based minimally invasive surgery training tools (12). Other toolkits, such as multimodal learning analytics, are being researched to potentially complement traditional measures of learning by providing high-frequency data on the behavior and cognition of trainees (112). Moreover, the integration of these technologies into daily surgical practice may negatively impact the skill development of surgical trainees. This issue should not be overlooked when leveraging AI's facilitative and enhancing effects on surgical education, and the surgical skill development of novice surgeons must be closely monitored (113, 114).

The trustworthiness of AI can be questionable in certain clinical settings (115). Although AI algorithms can rapidly analyze vast amounts of data, their reliability depends on factors such as the quality of the data, differences in patient populations, and other variables (110). Although AI systems in diagnostic settings may reduce interobserver variability, the training data must adequately represent the broader population (110, 116). To address this issue, AI systems should be trained using diverse and comprehensive datasets that accurately reflect the variability and characteristics of the broader population. On the other hand, there is a need for qualified personnel to implement these algorithms in surgical practice, because clinicians and healthcare professionals must be properly trained to use these systems effectively. Regular and continuous training programs should be developed to ensure that healthcare professionals receive adequate education on the use of rapidly evolving AI models (110).

Another set of challenges in implementing these technologies are ethical and legal considerations. These include the potential effects on data privacy and security as well as the challenges for regulating these ML-based surgical systems. The data used to train these algorithms often contain sensitive patient information, raising ethical concerns about privacy and security (110, 117, 118). A potential data leak could compromise patient confidentiality. To address these concerns, surgical and technological professionals must establish standards defining how and in which situations these technologies will be used. In addition, regulatory guidelines need to be developed and implemented simultaneously because these technologies continue to advance rapidly.

Another ethical consideration in using AI technologies in surgical settings is potential bias. Algorithms trained on biased datasets can reinforce healthcare inequalities related to race, sex, and socioeconomic status. Automation bias poses additional risks, particularly for under-resourced populations. To mitigate these issues, AI models should be developed and implemented with a focus on fairness and equity (118).

In addition to privacy and bias, a significant concern is the potential effect of AI's widespread use in modern medical practice, particularly in fields like radiology and pathology, for which it provides highly accurate diagnostic support (119). This potential raises concerns about job opportunities and employment in these areas, but it is important to remember that AI is a supportive tool in medical practice, not a replacement for human expertise (110).

Although challenges will surface when ML and hand-tracking technologies are developed and implemented, the potential for beneficial disruption in the surgical field is apparent. One exciting future trend will be integrating augmented reality and virtual reality technologies, which can potentially improve visualization during surgical skills training and intraoperative situations. In addition, advancements in sensor technologies and data fusion techniques will likely continue to improve upon the demonstrated accuracy of ML and hand-tracking models in both retrospective and real-time formats.

Ultimately, future research endeavors and the continued advancement of these technologies will be needed to address the current challenges. Nonetheless, ML and hand-tracking technologies can potentially have many exciting applications in the surgical field that should be considered. Future studies should focus on reducing the costs of applying AI systems in surgical practice, developing training programs to meet the demand for qualified personnel, and training AI algorithms with diverse datasets to enhance their applicability across various surgical disciplines (110). Emerging ML technologies like federated learning allow multiple devices or institutions to collaboratively train models while keeping their data private. The use of these recent AI models should be expanded comprehensively in future studies, with efforts focused on eliminating potential algorithmic biases and securing robust data privacy (120).

### 4.6 Limitations

This study aimed to collect the current literature on using diverse AI applications across various surgical settings involving hand or instrument tracking. Our primary goal was to create a resource for future studies rather than compare the effectiveness of different AI applications or ML paradigms. The heterogeneous nature of the data across the included studies represents a limitation, because differences in surgical instruments, settings, and methodologies make robust statistical comparisons challenging, and a risk of bias analysis was not conducted for the included studies.

## 5 Conclusions

In summary, ML and its subcategories, including supervised learning, unsupervised learning, reinforced learning, and deep learning, as well as the various sensor types such as optical, inertial, electromagnetic, ultrasonic, and hybrid sensors, offer unique strengths that could be combined and leveraged in different surgical training or patient care settings. The versatility of these technologies allows for the generation of adaptable models that cater to the specific surgical education and patient care questions that need to be addressed.

The hand-tracking technology and the applicability of ML have tremendous potential to grow, which leads to a direct, tangible impact in a wide range of sectors within surgery, such as surgical education of trainees and patient outcome management ranging from preoperative to postoperative stages. Acknowledging and tackling the current limitations of the development and use of ML will provide grounds for its successful optimization and application in the future.

Future studies that qualitatively and quantitatively depict the improvements generated by ML in surgical education and operative outcomes are warranted. A multifaceted approach to solving complex surgical problems in and out of the operating room should be the prioritized task to create novel solutions that complement and advance the delivery of patient-centered care.
